# Supplementary figures and images for: Redundant and specific roles of individual MIR172 genes in plant development
Source: PLoS Biol. 2021 Feb 2;19(2):e3001044. doi: 10.1371/journal.pbio.3001044 (PMC7853526; doi:10.1371/journal.pbio.3001044)

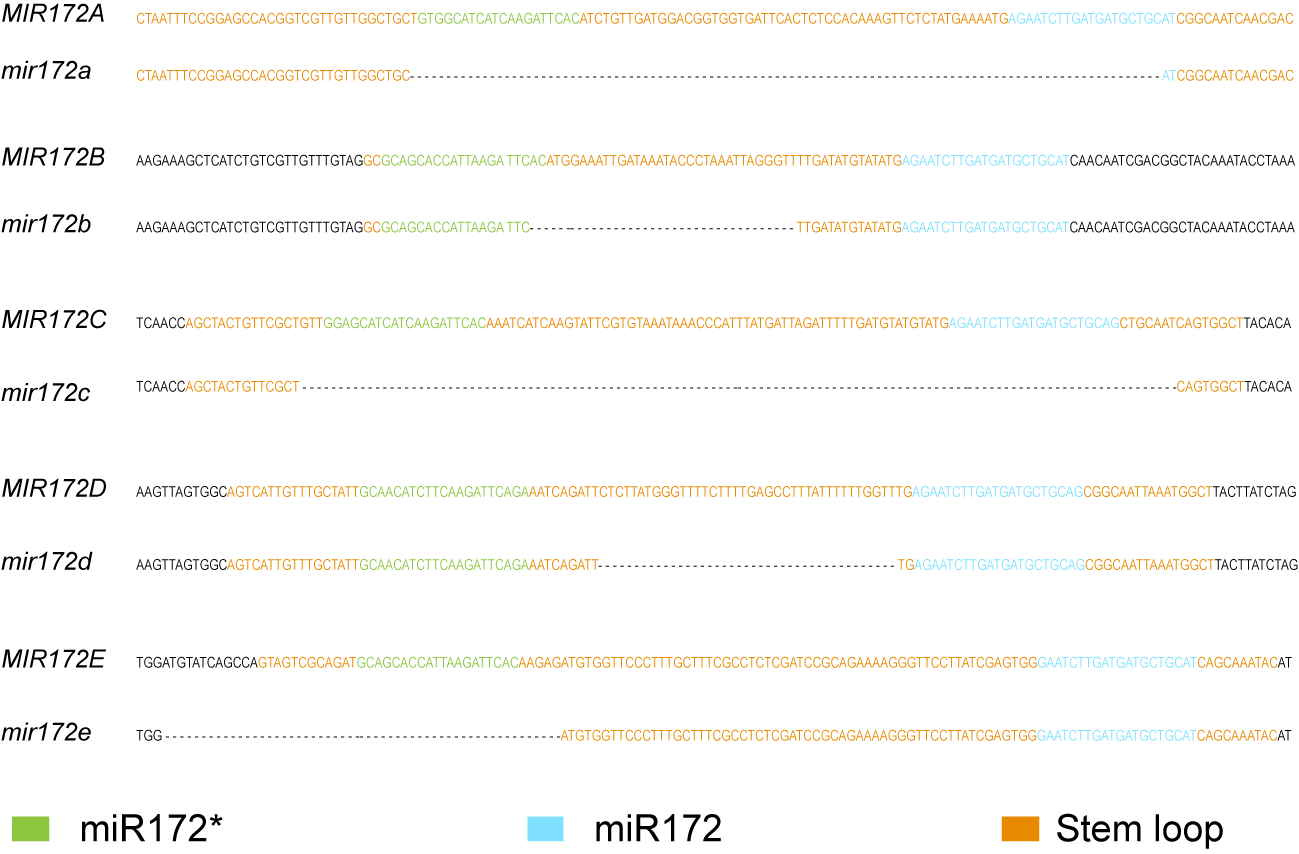

Supplement: S1 Fig — The genomic sequence of WT and mutated MIR172 loci. Orange, stem-loop of MIR172; green, miRNA*; blue, miRNA. CRISPR, clustered regularly interspaced short palindromic repeats; miRNA, microRNA; WT, wild type. (TIF) [file pbio.3001044.s001.tif]

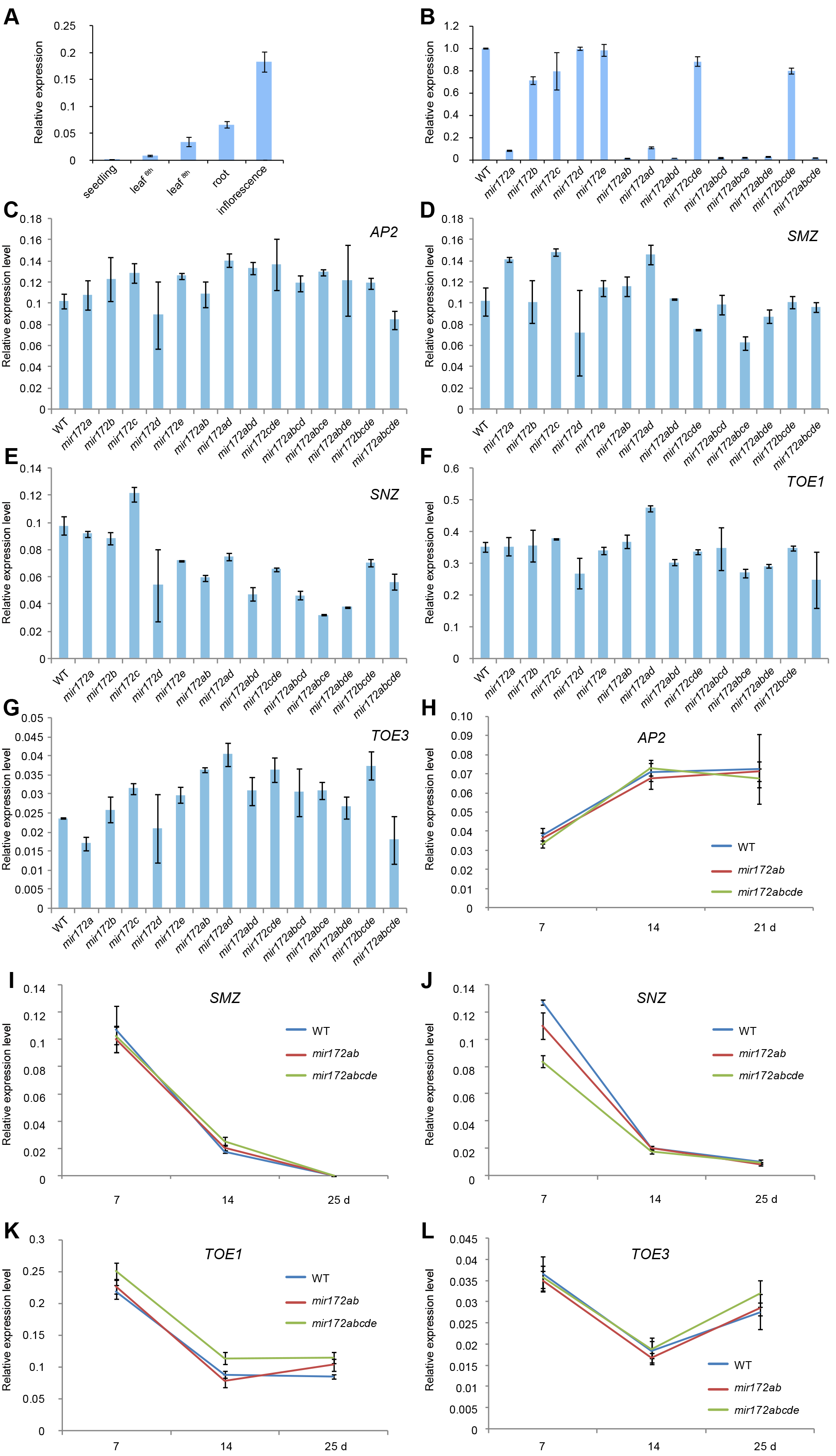

Supplement: S2 Fig — (A) Expression of miR172 in different tissues in WT. Seven-day-old seedlings, the 6th and 8th leaves, roots, and inflorescence were used. Two technical replicates for each biological replicate (n = 2) were performed. Error bars represent SD. (B) Expression of miR172 in the mir172 mutants. Twelve-day-old plants in long days were used for qRT-PCR analyses. Two technical replicates for each biological replicate (n = 2) were performed. Error bars represent SD. (C to G) Expression of miR172 targets in the mir172 mutants. Twelve-day-old plants in long days were used for qRT-PCR analyses. Two technical replicates were performed. Error bars represent SD. (H to L) Time course analysis of abundance of miR172 targets. Seven-day, 14-day, and 21/25-day-old plants in long days were used for qRT-PCR analyses. Two technical replicates for each biological replicate (n = 2) were performed. Error bars represent SD. For all the qRT-PCR assays, the expression level was normalized to that of TUB. The data underlying this figure are included in S2 Data. qRT-PCR, quantitative real-time PCR; TUB, β-TUBULIN-2; WT, wild type. (TIF) [file pbio.3001044.s002.tif]

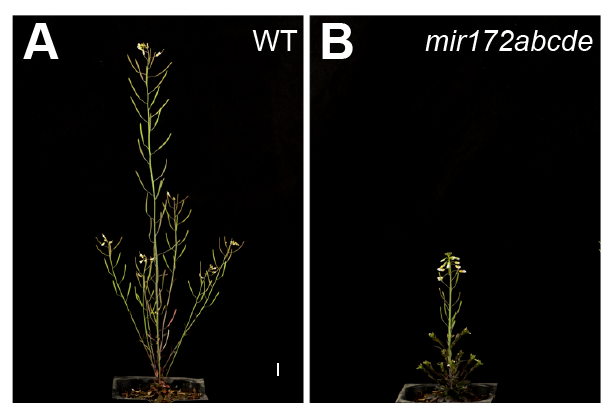

Supplement: S3 Fig — The photos were taken after plants flowered. Scale bar represents 1 cm. (TIF) [file pbio.3001044.s003.tif]

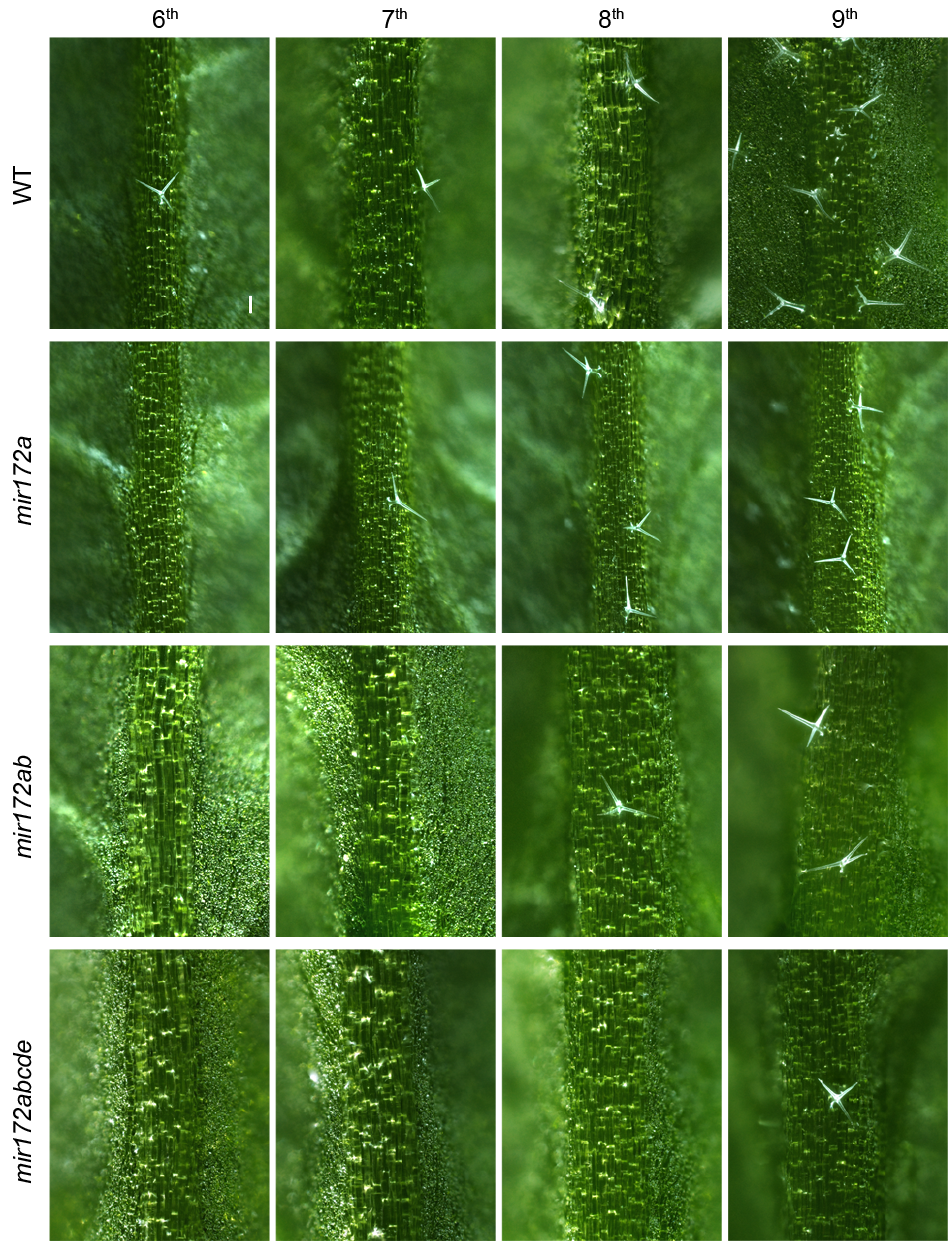

Supplement: S4 Fig — WT and the mir172 mutants were grown at 22°C in long days. The abaxial surfaces of the leaves are shown. Scale bar represents 200 μm. (TIF) [file pbio.3001044.s004.tif]

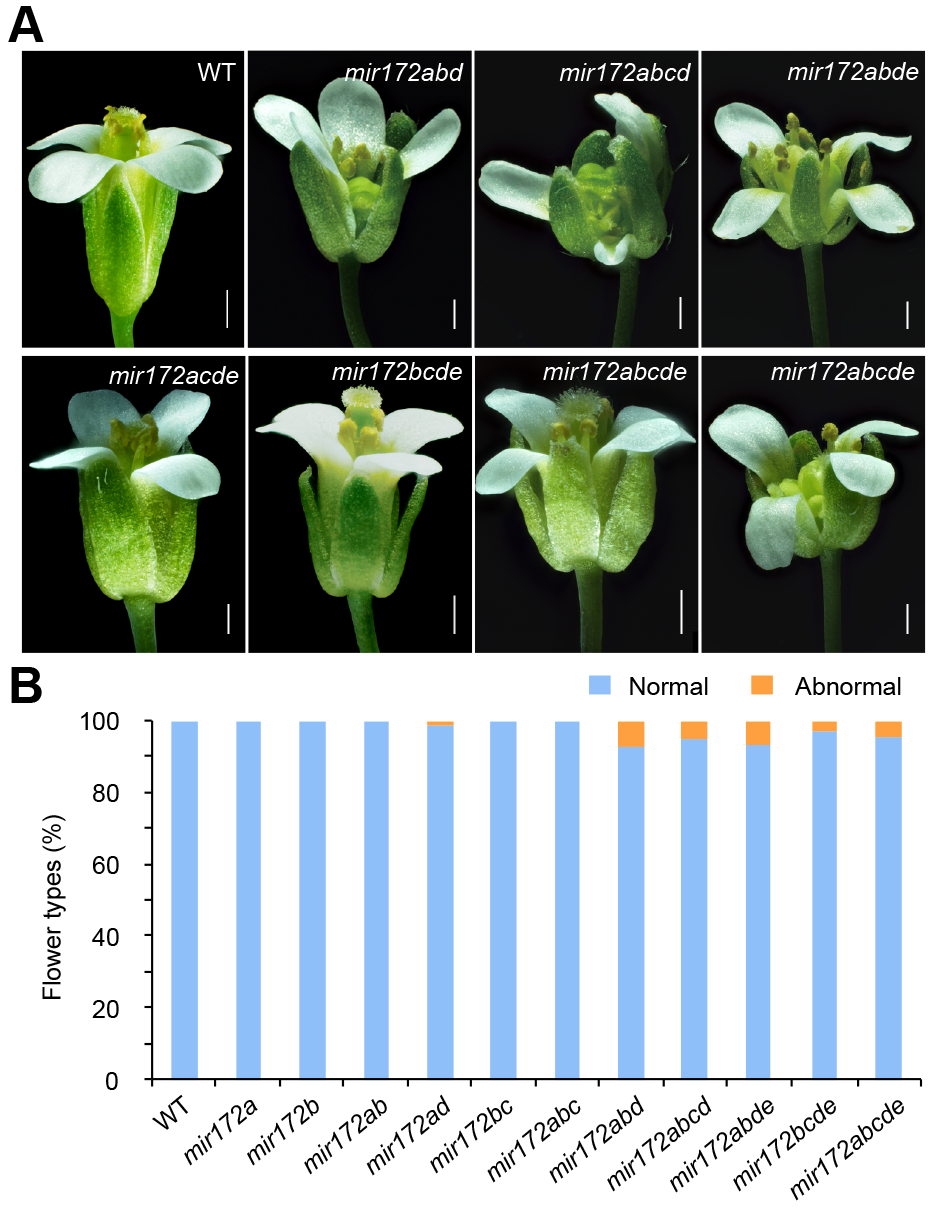

Supplement: S5 Fig — (A) The flowers of WT and the mir172 mutants. The flowers with abnormal phenotypes are shown. Scale bars represent 500 μm. (B) Quantification of abnormal floral phenotype in different genotypes. The floral phenotype of 5 to 21 plants for each genotype was examined. The data underlying this figure are included in S2 Data. (TIF) [file pbio.3001044.s005.tif]

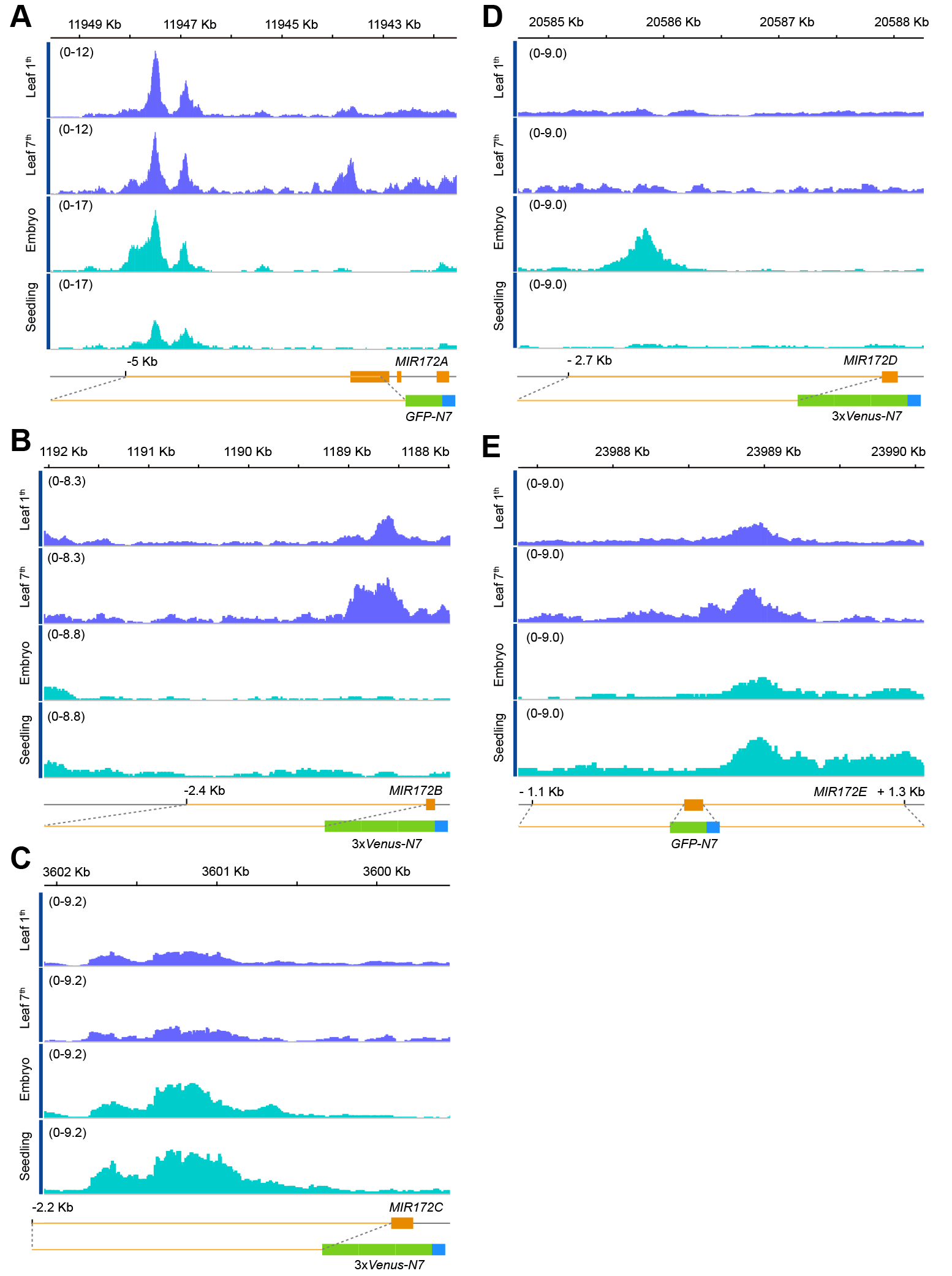

Supplement: S6 Fig — The ATAC-seq tracks for the MIR172 genes are shown. The datasets are derived from 4 plant tissues including the 1st leaf, the 7th leaf, embryo, and seedlings [56]. The orange box and line indicate stem loop region and the sequences used for generation of MIR172 reporter, respectively. For MIR172E reporter, the 1.3 kilobase pair (kb) downstream sequence was also included. The ATAC-seq datasets are deposited in Beijing Institute of Genomics Data Center (http://bigd.big.ac.cn) with the accession number (BioProject PRJCA002620 and BioProject PRJCA003872). The data underlying this figure are included in S2 Data. (TIF) [file pbio.3001044.s006.tif]

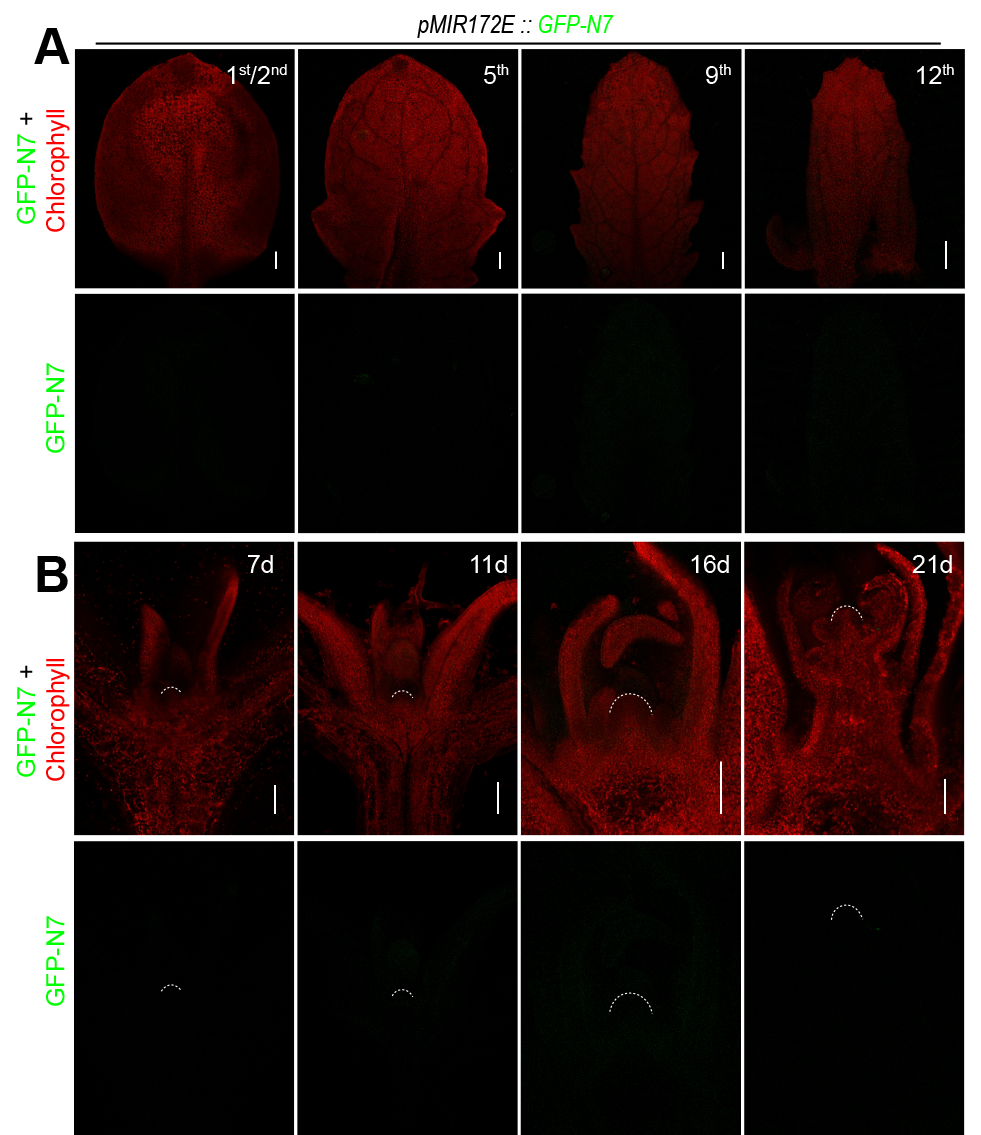

Supplement: S7 Fig — The leaves (A) and shoot apices (B) are shown. Plants were grown at 22°C in long days. The same pinhole size was used for scanning. Dash line marks the SAM. Scale bars represent 100 μm. (TIF) [file pbio.3001044.s007.tif]

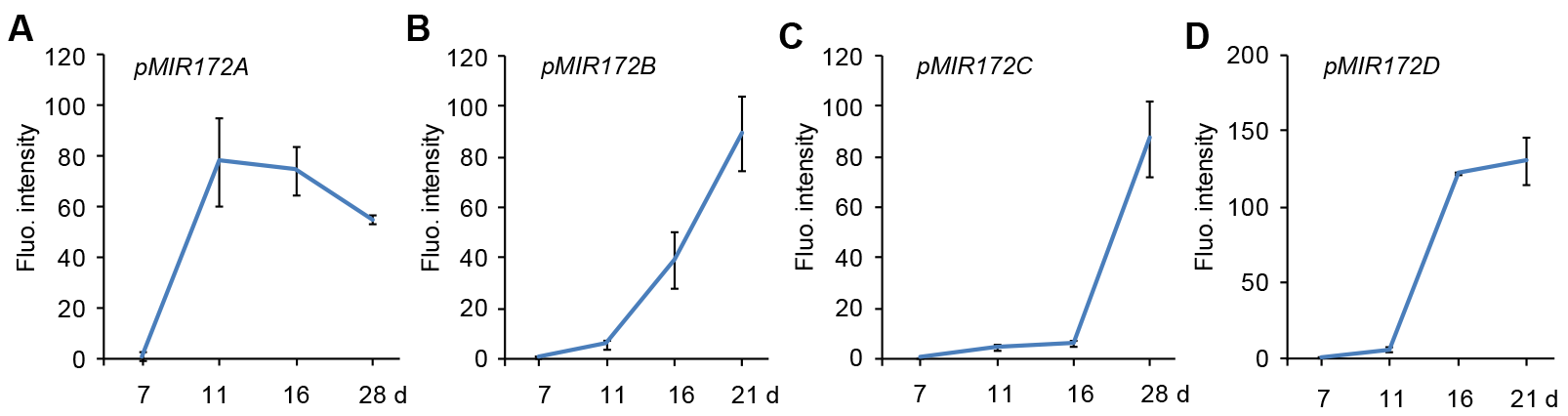

Supplement: S8 Fig — Plants were grown at 22°C in long days. See also Fig 4. The data underlying this figure are included in S2 Data. (TIF) [file pbio.3001044.s008.tif]

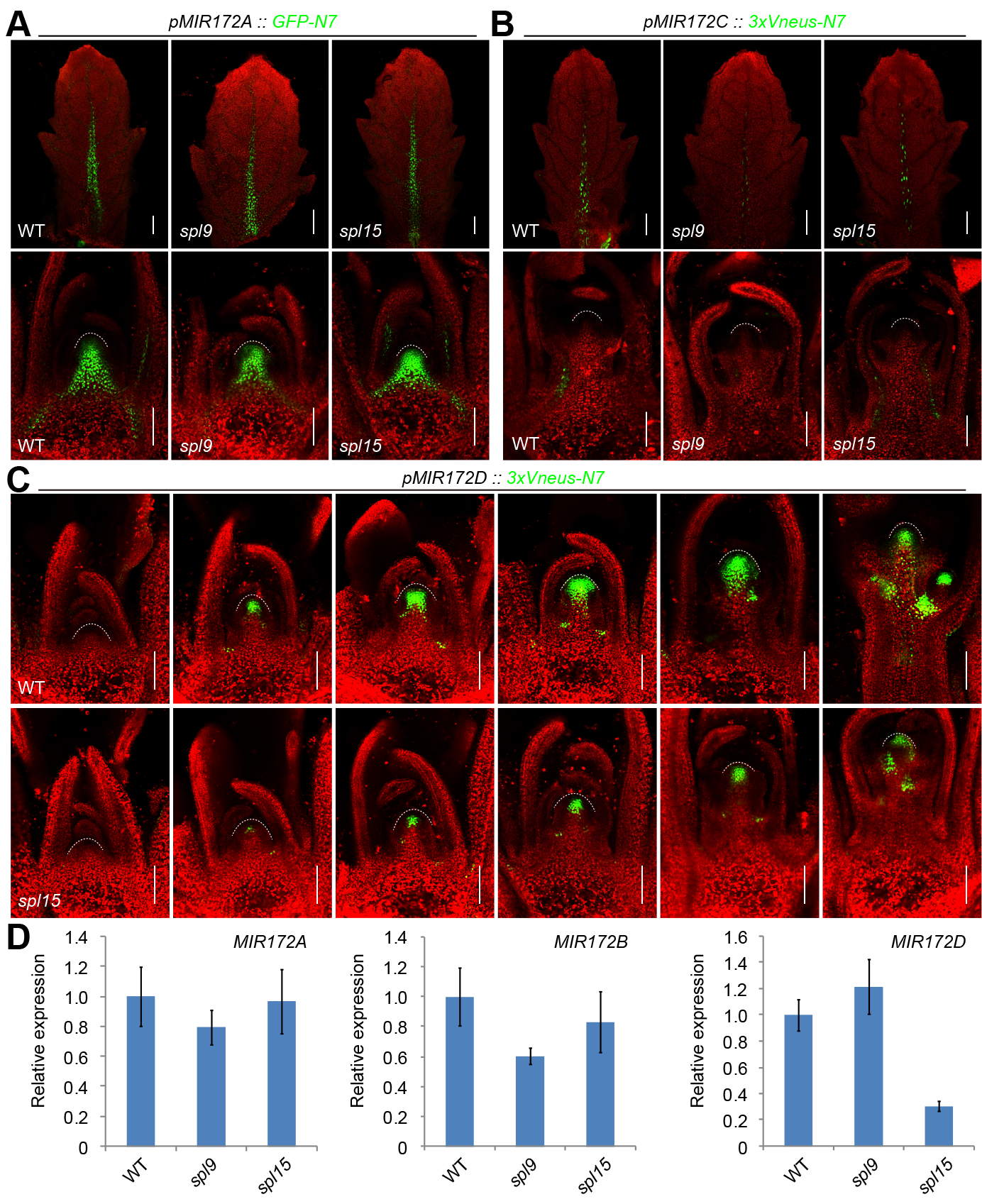

Supplement: S9 Fig — (A to C) Expression of MIR172 reporter genes in the spl9 and spl15 mutants. Plants were grown at 22°C in long days. Please note that the promoter activity of MIR172C was attenuated in the spl9 mutant but not in the spl15 mutant (B). In contrast, the transcription of MIR172A was largely unaffected by the mutation in SPL9 or SPL15 (A). The promoter activity of MIR172D was decreased in the spl15 mutant (C). The same confocal settings were used for scanning for each reporter line. Dash line marks the SAM. Scale bars represent 100 μm. (D) Expression of MIR172 genes in 18-day-old spl9 and spl15 mutants grown in LD. We harvested plants with the cotyledons and the first 5 rosette leaves manually removed. We could not get faithful data for MIR172C because its transcript level was very low. The expression level in WT is set to 1.0. Expression was normalized to TUB. Two technical replicates for each biological replicate (n = 2) were performed. Error bars represent SD. The data underlying this figure are included in S2 Data. LD, long days; SAM, shoot apical meristem; TUB, β-TUBULIN-2; WT, wild type. (TIF) [file pbio.3001044.s009.tif]

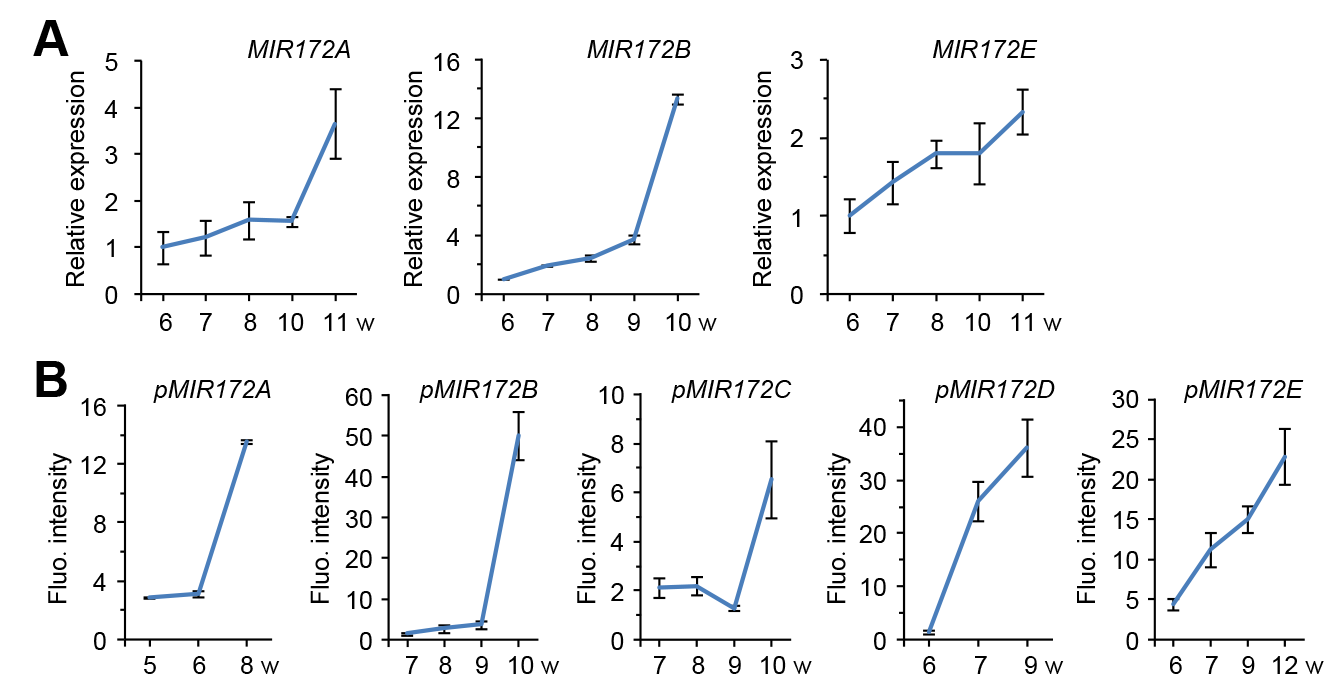

Supplement: S10 Fig — (A) qRT-PCR analyses of MIR172 genes. Due to low expression levels of MIR172C and MIR172D in short days, their expression were not faithfully quantified. Two technical replicates for each biological replicate (n = 2) were performed. Error bars represent SD. See also Fig 7. (B) Quantification of MIR172 reporter genes. See Fig 6. The data underlying this figure are included in S2 Data. (TIF) [file pbio.3001044.s010.tif]

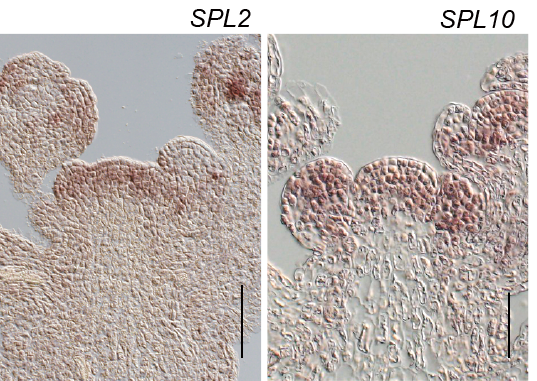

Supplement: S11 Fig — Scale bars represent 50 μm. (TIF) [file pbio.3001044.s011.tif]

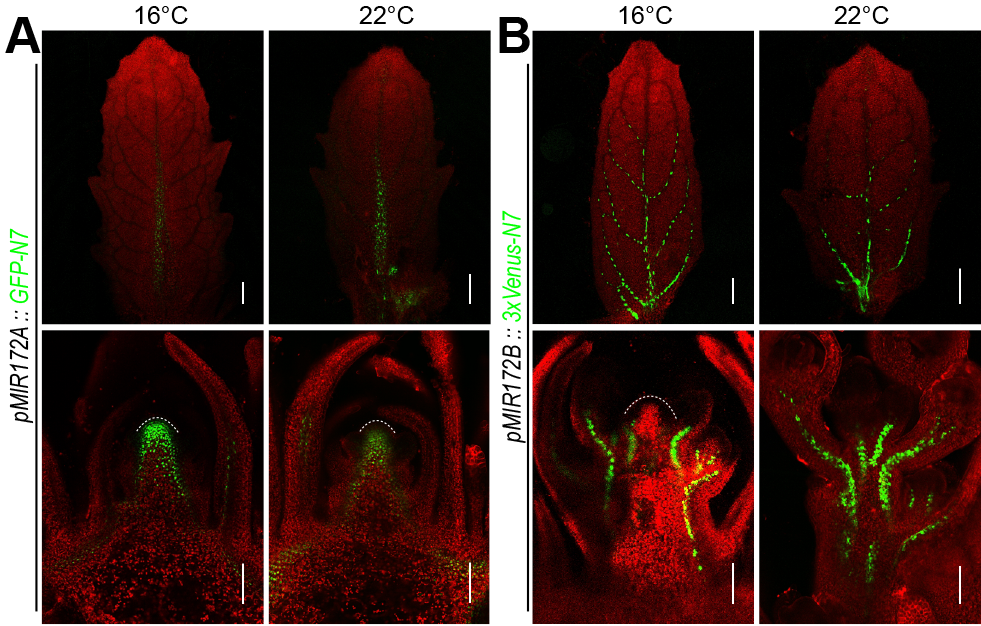

Supplement: S12 Fig — Expression of MIR172A (A) and MIR172B (B) in leaf and shoot apices. Plants were grown at 16°C or 22°C in long days. The same confocal settings were used for scanning for each reporter line. Dash line marks the SAM. Scale bars represent 100 μm. (TIF) [file pbio.3001044.s012.tif]

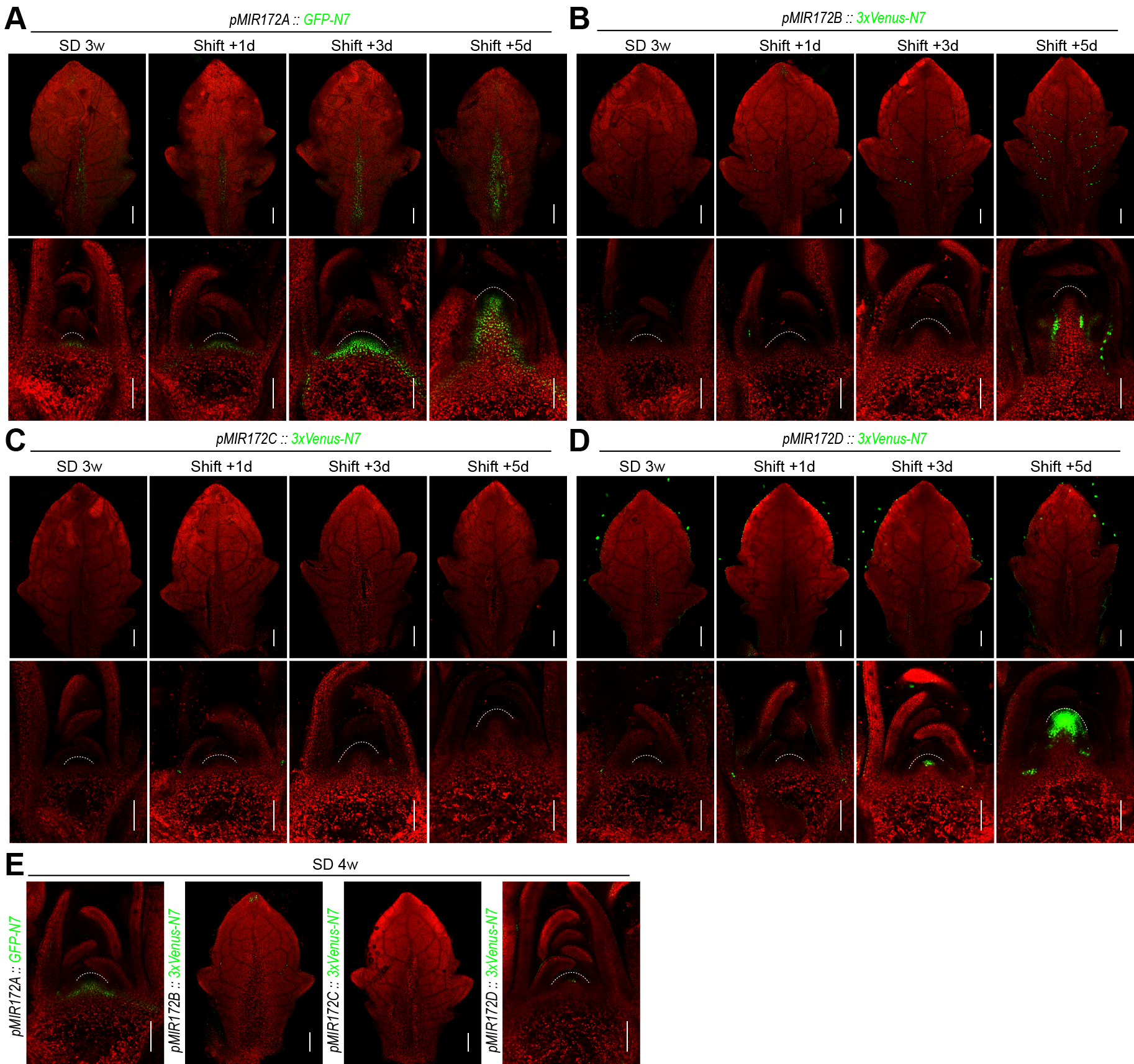

Supplement: S13 Fig — (A to D) The expression of MIR172 reporter genes before and after shift. Plants were grown in short days for 3 weeks (SD 3w) and shifted to long days (shift + n days). (E) The expression of MIR172 reporter genes after 4 weeks in short days (SD 4w). Please note that all the plants were still in the vegetative phase and the expression pattern of each reporter was the same as that of 3-week-old plants. The same confocal settings were used for scanning for each reporter line. Dash line marks the SAM. Scale bars represent 100 μm. (TIF) [file pbio.3001044.s013.tif]

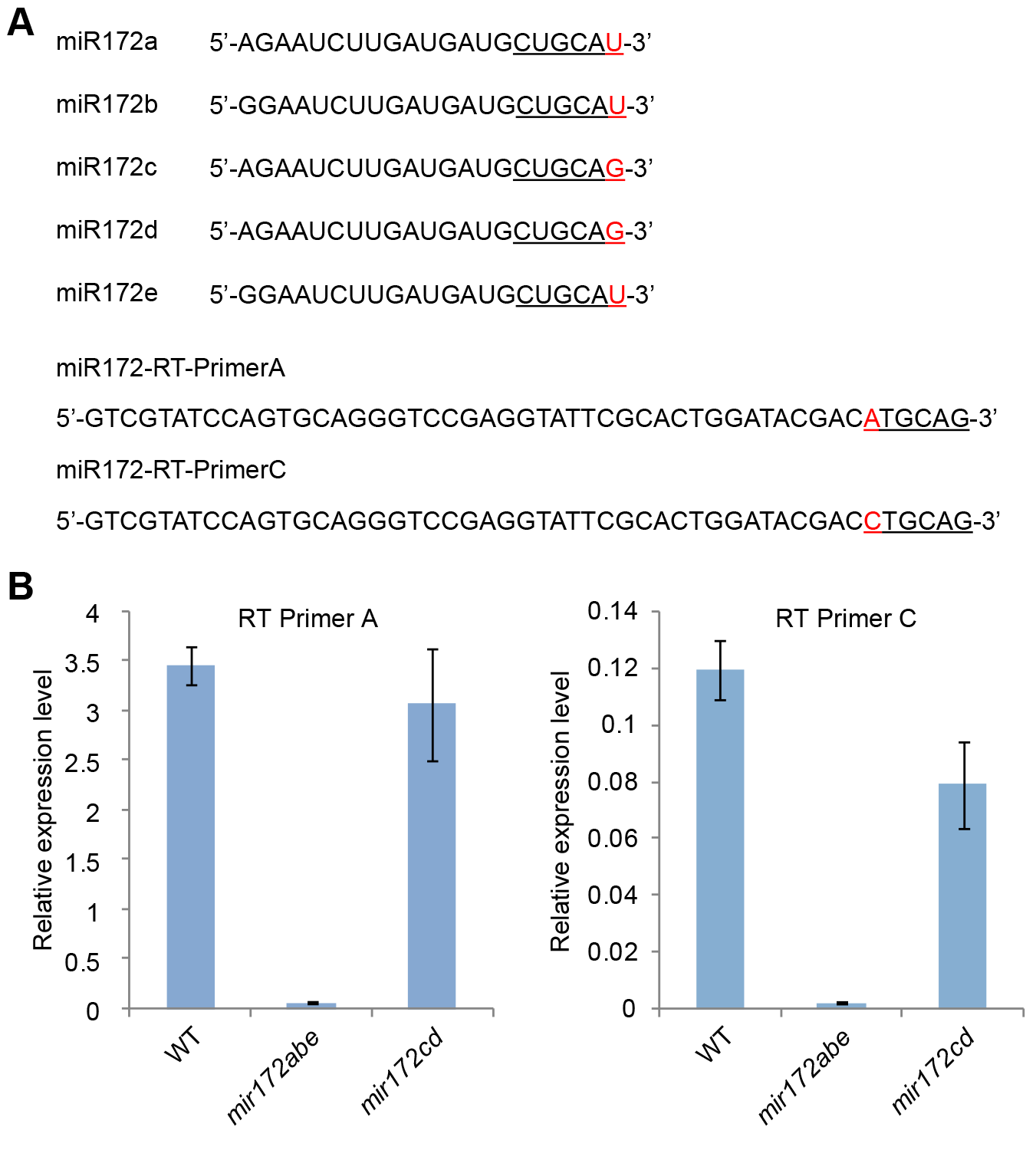

Supplement: S14 Fig — (A) The comparison of miR172 isoforms. The difference among miR172 isoforms resides in the last nucleotide (red). Two miR172-RT primers are designed: The miR172-RT-Primer A is fully complementary to miR172a, miR172b, and miR172e, whereas the miR172-RT-Prime C is fully complementary to miR172c and miR172d. These RT primers bind to the 3′ portion of miR172 molecules (underlined), initiating reverse transcription. (B) The expression of miR172 in WT and the mir172 mutants. We harvested 14-day-old plants grown in long days and set up 2 qRT-PCR experiments using miR172-RT-Primer A (left) or miR172-RT-Primer C (right) as the RT primer, respectively. Since MIR172D is highly expressed in the SAM, we could not detect its contribution to the mature miR172 pool. As such, the abundance of miR172 is markedly decreased in the mir172abe mutants. Both RT primers gave rise to similar results. However, the miR172-RT-Primer A gave higher reverse transcription efficiency than miR172-RT-Primer C. Notably, we could still detect miR172 in the mir172cd mutant using miR172-RT-Primer A as the RT primer, indicating that this primer does not discriminate miR172 isoforms. Expression was normalized to TUB. Two technical replicates for each biological replicate (n = 2) were performed. Error bars represent SD. The data underlying this figure are included in S2 Data. qRT-PCR, quantitative real-time PCR; SAM, shoot apical meristem; TUB, β-TUBULIN-2; WT, wild type. (TIF) [file pbio.3001044.s014.tif]
