# Supplementary material for: Redundant and specific roles of individual MIR172 genes in plant development
Source: PLoS Biol. 2021 Feb 2;19(2):e3001044. doi: 10.1371/journal.pbio.3001044 (PMC7853526; doi:10.1371/journal.pbio.3001044)
Supplement: Attachment — Submitted filename: Response to Reviewers.docx [file pbio.3001044.s021.docx]

Reviewers’ comments

**Reviewer # 1:**

This manuscript presents an analysis of redundant and/or specific functions of MIR172 gene family regulating plant development such as flowering time, axillary branching, meristem size and trichome development. The authors generate single mutants of each MIR172 A/B/C/D/E gene by CRISPR-Cas9 technology and analyze developmental phenotypes of a single mutant and higher-order mutants to address the redundant and/or specific functions of the each MIR172 gene. The authors further dissect the function of MIR172 genes in flowering time through genetic analysis using mir172 mutant plants and MIR172 gene promoter-driven reporter lines, showing that spatiotemporal expression of MIR172 gene family contributes to floral transition. Furthermore, in response to photoperiod, diversified function of MIR172 gene family is shown to be coupled with specific SPL gene possibly in a spatiotemporal specific manner.

Most of plant miRNAs are encoded by gene families but specific or redundant function of miRNA members has not been systemically addressed. miRNAs are thought to locally or distally act as signaling molecules and this study clearly shows that spatiotemporal activities of individual miR172 displays differential responses to developmental and environmental cues. Moreover, distinct pairs of SPL and miR172 regulate flowering time in response to photoperiod, suggesting that evolutionarily diversified miRNA family contribute to the internal and external signaling complexity in plants. This is a very well-organized and well-written paper, which can be a good example of how to study the diversified biological functions of plant MIRNA gene family. I have only a few minor comments.

Thanks for your supportive comments. We have revised the manuscript according to your suggestions.

1. How were the reporter lines in *spl9* and *spl15* (Fig. 4J and Fig. 6F) generated? Were they crossed to those mutants?

R: Yes, the reporter lines were crossed to the *spl9* and *spl15* mutant, respectively.

2. Following the first comments, it would be nice to determine expression levels of *MIR172A-E* in *spl9* (fig. 4J) and *spl15* (Fig. 6F) mutants to confirm the reporter line-based observation.

R: Done. The results are now given in S9D Fig. As you can see, *MIR172B* was reduced in the *spl9* mutant, whereas *MIR172D* expression was reduced in the *spl15* mutant. We could not quantify *MIR172C* probably because the processing of its primary transcripts is very fast.

**Reviewer # 2: Tony Millar – this reviewer has waived anonymity**

This is a very comprehensive study. Very impressively, they have used CRISPR to generate a quintuplet mutant in miR172, generating a very strong loss-of-function miR172 mutant that displays phenotypes they have not been previously observed from other approaches, such as using MIM172 transgenic plants. This has enable them to gain a greater insight into the role of miR172 in plant development, and has generated a very powerful genetic resource of mutant combinations to study the role of individual miR172 gene in the model plant Arabidopsis. The vast amount of data generated in this study has been very well presented into logical well thought-out figures. The paper gives insight into the contributions of these individual miR172 genes in different developmental processes, mainly that of the different cues of flowering-time.

Thanks for your positive comments. We have revised the manuscript according to your suggestions.

My major comment is more technical that that relating to the biology uncovered by the study. The structure of the reporter genes the authors made it not entirely clear to me. From Sup Fig 6, which covered the regions that were used in the construct, it seems that the stem-loop regions of the miR172 genes were also included, not just the 5' upstream regions (authors call this promoters in the Materials and methods). If these stem-loop regions are then in the same transcript as the reporter (GFP or Venus) would not then the transcript be processed by DCL1 and interfere with the reporter gene expression and potentially alter the pattern? I think this is an important consideration given the extent to which these reporters are used in the study.

R: I am sorry for this confusion. Actually, the stem-loop regions were not included in our reporters. We have clarified this point in S6 Fig.

Minor comments

Continuing on regarding the reporter lines, only one line per construct was examined in detail "We chose one representative T2 line for the subsequent analyses." - which "gave consistent and reliable expression patterns". Authors need to be clearer here, how do they know that the line picked was consistent and reliable? Does this mean of the 20 lines screened, all have highly similar expression patterns, so only one was chosen for detailed analysis? If so please state. As variation between lines is possible (as they are independent transgenic events), so was the one line chosen representative of the general expression pattern observed most of the 20 lines? i.e. for MIR172d -did a majority of the 20 share the observation that they are expressed in leaf margins. If this is the case, I think the authors need to add a few words for a clearer explanation. If not this would also be of concern.

R: The T1 transgenic lines were screened on soil with BASTA, and individual plants (>=20 lines) were examined under BX63 fluorescent microscope (Olympus). All the examined plants exhibited similar expression pattern, although the intensity of the GFP/Venus signals varied. The seeds of the T1 lines with strong fluorescent signal were collected and sowed on soil with BASTA, and the single copy insertion lines (by determining the separation ratio) were used for subsequent analysis.

For *pMI172A::GFP-N7* and *pMIR172D::3xVenus-N7* reporters, the GFP or VENUS fluorescence in the IM were dissected and observed under the BX63 fluorescent microscope (Olympus). Similarly, for *pMIR172B::3xVenus-N7* and *pMIR172C::3xVenus-N7* reporters, the VENUS fluorescence of the IM and stem were examined. For *pMI172E::GFP-N7* reporter, GFP fluorescence of the IM and roots were examined.

Conflicting statements that need to be clarified "the five MIR172 gene family members are functionally redundant but exert individual specificities in regulating diverse aspects of plant development" - but in the discussion - "we have demonstrated that the Arabidopsis MIR172 gene family members are definitely not redundant" - think the former statement is more accurate and the latter misleading - they may not be fully redundant, but they play overlapping roles in some instances. Please clarify.

R: We fully agree with this argument. We changed this sentence into “In this study, we have demonstrated that the Arabidopsis *MIR172* gene family members are not only redundant but also confer functional specification (Fig 2I). Our reporter analyses further reveal that this specificity can be attributed to their distinct expression pattern, in addition to variations in miRNA processing efficiency.”.

Figure 3C. Here it appears mature miR172 levels are normalized to TUB, a mRNA not a small RNA?? Also, miR172a and b, are the same isoform, whereas the others have sequence variations. Are the primers used preferentially detect the a/b isoform and therefore skew the data. Need more information here, the isoforms, the primer used (any mis-matches with c, d and e?), and why TUB was used (polyA synthesis?) versus small RNA Reverse Transcription with a specific primer??

R: We used the method according to Varkonyi-Gasic et al (Plant Methods, 2007). In this protocol, miRNA and mRNAs can be simultaneously reverse-transcribed and subjected to downstream analysis.

In addition, based on its principle, this qRT-PCR protocol does not discriminate different miRNA isoforms within a gene family. The difference among miR172 isoforms resides in the last nucleotide (red, S14A Fig). We designed two miR172-RT primers: The miR172-RT-Primer A is fully complementary to miR172a, miR172b and miR172e, whereas the miR172-RT-Prime C is fully complementary to miR172c and miR172d. These RT primers bind to the 3' portion of miR172 molecules (underlined), initiating reverse transcription (S14A Fig).In the first version of our manuscript, we used miR172-RT-Primer A as the RT primer.

We setup two qRT-PCR experiments using miR172-RT-Primer A (left) or miR172-RT-Primer C (right) as the RT primer respectively (S14B Fig). The miR172-RT-Primer A gave a higher reverse transcription efficiency than miR172-RT-Primer C. However, both RT primers gave rise to similar results. We could still detected miR172 in the mir172cd mutant using miR172-RT-Primer A as the RT primer (left, S14B Fig), indicating that this miR172-RT-Primer A does not discriminate miR172 isoforms.

**S14 Fig. Validation of qRT-PCR primers**

Ref: Varkonyi-Gasic E, Wu R, Wood M, Walton EF, Hellens RP. 2007. Protocol: a highly sensitive RT-PCR method for detection and quantification of microRNAs. Plant Methods. 3:12. doi: 10.1186/1746-4811-3-12.

Phrases/ typos

Page 3, line 11; *MIR319* should be *MIR159* as indicated in the next sentence.

R: Done.

Page 5 line 19 and other places; Using the word "coding" regarding a miRNA gene could be regarded as incorrect given they are usually classified as "non-coding RNA". E.g. "the entire stem-loop regions of the "coding" genes" - maybe better to say "MIR172 genes"

R: Done. We changed “*MIR172* coding genes” into “*MIR172* genes”.

**Reviewer # 3:**

**Revision of "Redundant and Specific Roles of Individual MIR172 Coding Genes in Plant Development" by Heng Lian et al.**

This work analyzed the *MIR172* family of small RNAs in *Arabidopsis thaliana*, which is comprised by five members. The authors used CRISPR-Cas9-induced mutations to inactivate each MIR172 family member. The authors characterize diverse phenotypic defects after the inactivation of different combinations of MIR172 genes, including the modification of meristem size, trichome initiation, stem elongation, shoot branching and floral competence. Furthermore, the authors demonstrate the role of specific SPL-MIR172 pairs in different tissues.

The generation of mutants for all MIR172 family members and the thorough and laborious genetic analysis performed here are very well done and I think that the results will be a of wide interest to the scientific community. Overall, the results are presented in a clear way highlighting the role of MIR172s as a hub for the integration of different signals.

Thanks for your positive comments. We have revised the manuscript according to your suggestions.

My main comment about the manuscript is that it is lacking a concomitant description about the expression miR172-targets in WT and *mir172* mutants. At least, the expression of miR172-targets could be determined by RT-qPCR in the samples described in Figure 3C or S2B.

R: We added the requested qRT-PCR results in Fig 3C and S2B Fig. Consistent with a previous report from Xuemei Chen lab (Chen, Science, 2003), we also found that miR172 represses its targets mainly by translational inhibition. As such, the transcript levels of miR172 targets did not greatly changed in the *mir172* mutants (S2C to S2L Fig2).

Additional comments:

1) The reporters for MIRNA expression were designed by replacing the precursor sequence with a reporter. I wonder whether this strategy could result in a reporter mRNA having long 5' UTR with spurious ATGs that will in turn affect its expression. Please comment.

R: Actually, this is a very good question. We don’t have answer yet. However, based on literatures (miR159, Allen et al., PNAS, 2007; miR160, Wang et al., TPC, 2005; miR171, Han et al., Nat Commun, 2020), it seems that the spurious ATGs in the *MIRNA* promoter do not affect reporter expression.

2) Title "MIR172 coding genes". I think it should be "miR172-coding genes". Anyways, I'm not sure whether it is convenient to use "coding".

R: Thanks for your suggestions. We changed “*MIR172* coding genes” into “*MIR172* genes”.

3) Introduction: "Due to the small sizes of the genes, simultaneous inactivation of all miRNA family members by generation of multiple transfer-DNA (T-DNA) mutant lines has so far been achieved for only two relatively small families, MIR164 (i.e. MIR164A-C) and MIR319 (i.e. MIR319A-C) [11, 12]" -- It is MIR59A-B instead of MIR319A-C.

R: fixed.

4) Introduction: "These two studies showed that, similar to protein-coding genes, the MIRNA coding genes in the same family are functionally redundant." -- MIR164C (early extra petals1) has also specific functions (Baker et al. Curr Biol 2005)

R: Thanks for your suggestion. We add this in the Discussion (Page 13).

5) I think Figure S1 is nicer than Figure 1. Perhaps the authors might want to move Figure S1C to Figure 1.

R: Thanks for your suggestion. We have revised Fig 1 and S1 Fig accordingly.

6) Page 13 l6 "promoting the procession" - I think there is a typo "promoting the processing"

R: Fixed.
